# Acromioclavicular joint dislocation: a comparative biomechanical study of the palmaris-longus tendon graft reconstruction with other augmentative methods in cadaveric models

**DOI:** 10.1186/1749-799X-2-22

**Published:** 2007-11-27

**Authors:** Guntur E Luis, Chee-Khuen Yong, Deepak A Singh, S Sengupta, David SK Choon

**Affiliations:** 1 Department of Orthopaedics Surgery, University of Malaya, Kuala Lumpur, Malaysia

## Abstract

**Background:**

Acromioclavicular injuries are common in sports medicine. Surgical intervention is generally advocated for chronic instability of Rockwood grade III and more severe injuries. Various methods of coracoclavicular ligament reconstruction and augmentation have been described. The objective of this study is to compare the biomechanical properties of a novel palmaris-longus tendon reconstruction with those of the native AC+CC ligaments, the modified Weaver-Dunn reconstruction, the ACJ capsuloligamentous complex repair, screw and clavicle hook plate augmentation.

**Hypothesis:**

There is no difference, biomechanically, amongst the various reconstruction and augmentative methods.

**Study Design:**

Controlled laboratory cadaveric study.

**Methods:**

54 cadaveric native (acromioclavicular and coracoclavicular) ligaments were tested using the Instron machine. Superior loading was performed in the 6 groups: 1) in the intact states, 2) after modified Weaver-Dunn reconstruction (WD), 3) after modified Weaver-Dunn reconstruction with acromioclavicular joint capsuloligamentous repair (WD.ACJ), 4) after modified Weaver-Dunn reconstruction with clavicular hook plate augmentation (WD.CP) or 5) after modified Weaver-Dunn reconstruction with coracoclavicular screw augmentation (WD.BS) and 6) after modified Weaver-Dunn reconstruction with mersilene tape-palmaris-longus tendon graft reconstruction (WD. PLmt). Posterior-anterior (horizontal) loading was similarly performed in all groups, except groups 4 and 5. The respective failure loads, stiffnesses, displacements at failure and modes of failure were recorded. Data analysis was carried out using a one-way ANOVA, with Student's unpaired t-test for unpaired data (S-PLUS statistical package 2005).

**Results:**

Native ligaments were the strongest and stiffest when compared to other modes of reconstruction and augmentation except coracoclavicular screw, in both posterior-anterior and superior directions (p < 0.005).

WD.ACJ provided additional posterior-anterior (P = 0. 039) but not superior (p = 0.250) stability when compared to WD alone.

WD+PLmt, in loads and stiffness at failure superiorly, was similar to WD+CP (p = 0.066). WD+PLmt, in loads and stiffness at failure postero-anteriorly, was similar to WD+ACJ (p = 0.084).

Superiorly, WD+CP had similar strength as WD+BS (p = 0.057), but it was less stiff (p < 0.005).

**Conclusions and Clinical Relevance:**

Modified Weaver-Dunn procedure must always be supplemented with acromioclavicular capsuloligamentous repair to increase posterior-anterior stability. Palmaris-Longus tendon graft provides both additional superior and posterior-anterior stability when used for acromioclavicular capsuloligamentous reconstruction. It is a good alternative to clavicle hook plate in acromioclavicular dislocation.

## Introduction

The acromioclavicular and coracoclavicular ligaments of the shoulder joints are prone to sports injuries especially in throwing athletes. The mechanism of injury usually involves a direct trauma to the superior aspect of the acromion and includes inferior and anterior translation of the acromion in relation to the distal aspect of the clavicle. Operative treatment has been advocated for certain type III acromioclavicular joint separations and certainly in types IV and V acromioclavicular joint injuries [1-3,12,13,20]. Previous studies have demonstrated that the acromioclavicular ligaments control anterior-posterior stability, while the coracoclavicular ligaments control superior-inferior stability [16,27].

The original Weaver and Dunn technique, first described in 1972, did not include augmentation device [8,29]. Later studies showed results in favour of augmenting the strength of the coracoacromial ligament transfer while it is healing [6,10,14,15,22,25]. Current operative techniques can be classified into 2 groups : 1) Those that focus on the primary healing of the coracoclavicular ligaments, by holding the clavicle and coracoid in a reduced position and 2) those that focus on reconstructing the coracoclavicular ligaments, using local tissue transfers or tendon grafts. The former allows primary healing of the coracoclavicular ligament by either fixing the acromioclavicular joints using K-wires, Steinman pins, tension banding, and clavicle hook plates or fixing the coracoid to the clavicle using screws, sutures, suture anchors, tapes and direct suture of the coracoclavicular ligaments. These techniques assume that the coracoclavicular ligaments will heal at its near preinjury tensile strength. The latter transfers local tissue sources to the clavicle or uses tendon grafts, either autografts or allografts. One common problem with these techniques remains the weak initial fixation of the ligament or tendon to the clavicle.

There is an increasing trend in using tendon grafts for reconstructing the coracoclavicular ligaments. We have chosen a novel reconstruction technique for the acromioclavicular capsuloligamentous complex using the palmaris-longus tendon graft since the palmaris-longus tendon is dispensable and can be harvested with low morbidity. The objective of this study is to compare the biomechanical properties of this novel palmaris-longus tendon reconstruction with those of the native AC+CC ligaments, the modified Weaver-Dunn reconstruction, the ACJ capsuloligamentous complex repair, screw and clavicle hook plate augmentation.

## Methods

### Sampling

56 fresh frozen shoulders were obtained from unclaimed bodies. Two shoulder specimens were excluded because of gross comminuted scapula fractures. The ages of the specimens ranged from 25 to 46 years old, with a mean of 35+/-11 years old. There were 27 right and 27 left shoulders. There were 10 pairs of female and 17 pairs of male shoulders. There was no gross pathology of the ligaments or bones. None of the shoulders had been previously operated on. The glenohumeral and sternoclavicular joints were disarticulated. The shoulders were dissected free of all skin, muscle and subcutaneous tissues. The clavicles and scapulae were exposed, carefully preserving the acromioclavicular (ACL) and coracoclavicular (CCL) ligaments. No prior sectioning of these ligaments was done to allow accurate simulation of the non-selective nature of clinical ligament injury.

The coracoacromial ligaments were resected at its insertion on the undersurface of the acromion, prior to testing. This removes any confounding effects since the coracoacromial ligaments, often blending in with the inferior acromioclavicular ligaments, may exert an inferior restraining force. No distal clavicle end resection was performed. The specimens were stored at -20 deg. Before the day of the test, each shoulder specimen was thawed overnight at room temperature.

The 54 grossly normal fresh frozen shoulders were tensile tested to failure, using the Instron Machine Model 8846, to compare the structural properties of the i) combined native acromioclavicular and coracoclavicular ligaments, ii) the coracoacromial ligament transfer in modified Weaver-Dunn reconstruction, iii) efmodified Weaver-Dunn reconstruction with the acromioclavicular capsuloligamentous repair, iv) modified Weaver-Dunn reconstruction with the coracoclavicular screw augmentation, v) modified Weaver-Dunn reconstruction with clavicle hook plate augmentation and vi) modified Weaver-Dunn reconstruction with ACJ reconstruction using palmaris-longus tendon graft and mersilene tape augmentation. At a crosshead speed of 50 mm per min, the specimens were tested for superior and anterior displacements. This low crosshead speed used because failure occurs at both a higher load and greater extension if the test is done at high speed, which means that more energy is needed to rupture the specimen at high speed. Stiffening effect of the ligaments could also be minimized at this low rate. Pretensioning was performed at 70 N (physiological load) to reduce the "crimp" effect of the ligaments to straighten the collagen fibres.

The acromioclavicular joint is a true diarthrodial joint formed by the articular surfaces of the outer end of the clavicle and of the acromion. The clavicle and acromion are united by a capsule inserting a few millimeters from the articulating surfaces. This loose capsule is reinforced on the superior and inferior aspect by the powerful acromioclavicular ligament which runs transversely over the joint. The superior component is much better developed and thicker than the inferior acromioclavicular ligament. A resultant force causing ligament failure can be resolved into 3 vectors in the x, y and z axes. The magnitude of a force required to disrupt the abovementioned transverse fibres is the least when applied in a direction perpendicular to the direction of these fibres, as compared to when the force is directed parallel to the direction of these fibres.

**Figure 1:**
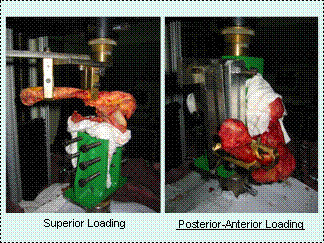
Test Rig Setup.

The setup of the test rig (Fig. 1), was therefore designed to apply these perpendicular forces to the fibres, in the superior and anterior directions (2 axes). These forces were the most common disruptive forces in injuries. The 3^rd ^axis (distractive force parallel to the direction of the fibres and long axis of the clavicle) subjecting the AC joint to distractive force is not tested since it is uncommon. The anatomical position was defined by aligning the bony articulation of the distal end of the clavicle and the acromion process, with equal tensioning throughout the soft tissue structures. Custom-made clamps were used to mount the clavicle to the crosshead and the scapula to the base of the Instron machine such that a load as perpendicular as possible can be applied. The long axis of the clavicle and the scapular plane were oriented at approximately 90 degrees to one another. To ensure that the coracoclavicular ligament complex is centered under the crosshead, one clamp is placed medially to the CC ligament, while the other is placed in between the CC and AC ligament complexes.

This testing setup assumed that in an ACJ dislocation injury, there was no movement in the sternoclavicular joint (ie, the clavicle and sternum acted as one unit). The values for loads to failure, obtained for this study, were thus the least forces required for ACJ dislocation in the particular direction of interest.

The acromial reference point was defined as the centroid of its surface. With the aid of a proportional divider, the medial boundary of the acromion was determined. The two most anteromedial and posteromedial points of the acromion were then established. A line A, connecting these two points, was drawn and its length measured using a caliper. Line B, with length b, was constructed perpendicularly from line A to the medical concave aspect of the acromion. The medial concave aspect of the acromion, articulating with the lateral end of the clavicle, most closely approximated the arc of a semi-ellipse. The centroid of acromion, coordinate (X, Y), was thus outside the acromion. The midpoint of line A was taken as the mean of all X of the acromion. The mean of all Y for the acromion was described by the formula 4b/(3 × 3.14). If the distance b is zero, then the acromion was in total contact with the lateral end of the clavicle. [See Additional file 1]

The distal clavicular reference point was defined as the point on the clavicle in contact with the acromial reference point in the intact, unloaded joint. The joint separation, in response to a known applied load, was determined along 2 axes. The posterior-anterior displacement was defined as the distance between the point of maximum anterior displacement of the clavicle reference point and the neutral position of the clavicle reference point (corresponding to the application of the 100-N force anteriorly). The superior displacement was defined as the distance between the point of maximum superior displacement of the clavicle reference point and the neutral position of the clavicle reference point.

Increasing load was then applied to each specimen until the testing endpoint was achieved, that is complete tear of ACJ and ligament, complete failure of ligament reconstruction or complete failure of reconstruction-augmentation construct and specimen failure. Superior displacement in the coronal plane and anterior displacement in the sagittal plane were determined by measuring joint separation as the clavicle was loaded in the superior and anterior directions respectively. There was no movement between the clamps and specimens during testing. The movement from the AC joint was equal to the displacement of the load cell and recorded simultaneously by the Instron machine software, as the loads were being generated. Parallel reference indicators (linear frames with accuracy to 0.1 cm) attached to either side of the load cell also allows measurement of separation, with error of +/- 6%. The respective failure loads, displacement at failure, stiffness and modes of failure were recorded. When "failure" status was reached, the load-cell returned the clavicle to its original pre-tensioned resting position, with respect to the acromion, as preset in the software program. Unless a fracture or deformation occurred, the same scapula and clavicle was used for each of the subsequent reconstructions.

The order of testing sequence was not randomized and executed in the following manner:

Testing Sequence

(1) Superior Loading.

Native Lig → WD → End Point (38 Specimens).

→ WD + ACJ → End Point (9 Specimens).

→ WD + CP → End Point (10 Specimens).

→ WD + BS → End Point (10 Specimens).

→ WD + PL-MT → End Point (9 Specimens).

(2) Posterior-Anterior Loading.

Native Lig → WD → End Point (16 Specimens).

→ WD + ACJ → End Point (8 Specimens).

→ WD + PL-MT → End Point (8 Specimens).

("→ " implies tested to failure)

### Reconstruction and Augmentation Techniques

• Modified Weaver-Dunn reconstruction.

**Figure 2:**
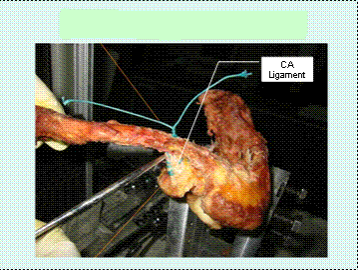
Modified Weaver-Dunn reconstruction.

The modified Weaver-Dunn reconstruction (Fig 2) was performed by dividing the coracoacromial ligament at its acromial insertion. The freed acromial end of the coracoacromial ligament was anchored with whipstick sutures using No.2 Ethibond sutures (Johnson and Johnson). Prior templating of the future 3.5 mm drill-hole sites was made with the clavicle hook plate sitting on the superior aspect of the clavicle. The stump of the coracoacromial ligament was drawn into one of the middle drill-holes through the inferior cortex and out of the superior cortex of the clavicle. The sutures were then tied around the anterior half of the clavicle. Repair of the acromioclavicular capsuloligamentous complex was performed using Bunnell-type weave with No. 2 Ethibond suture. The distal ends of the clavicles were not resected to allow for repair of the acromioclavicular capsuloligamentous complex and optimal plate sitting on the clavicle.

• ACJ capsuloligamentous repair.

The acromioclavicular capsuloligamentous complex using a Bunnell-type weave with No 2 Ethibond sutures.

• Clavicle hook plate augmentation.

**Figure 3:**
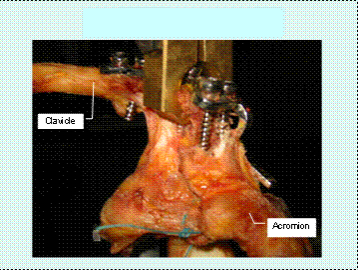
Clavicle hook plate augmentation.

The acromioclavicular joint was reduced under vision. The clavicle hook plates, (Fig 3), with 6 or 8 holes, are precontoured in left and right plates. They are available in commercially pure titanium and stainless steel. The hook of the plate (Synthes) with a 15 mm or 18 mm hook depth was first passed under the acromion, then on the superior aspect of the clavicle. Finally, 3.5 mm cortical screws were placed in the medial and anterolateral screw holes. The coracoacromial ligament graft can be tunneled into one of the middle screw holes of the plate. The plate with 18 mm hook depth is used instead if there is difficulty lowering the plate shaft onto the clavicle.

Its use is especially advantageous in situations where concomitant coracoid process fracture precludes the use of bioabsorbable tape slings or coracoclavicular screw fixation.

**Figure 4:**
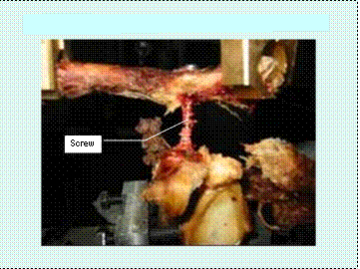
Coracoclavicular Screw augmentation.

• Coracoclavicular screw augmentation (Fig 4).

A modification of the method described originally by Bosworth was performed. [2] The AO cortical screw (Synthes) was positioned starting from the posterior part of the clavicle 4 cm from its lateral end and passing forward and downward to be inserted into the base of the coracoid process. A 4.5 mm hole was first drilled in the clavicle and then a 3.2 mm drill, passing through this hole, advanced into the base of the coracoid. A 4.5 mm AO screw of adequate length, with a large washer, was now inserted through the hole and screwed into the coracoid until the clavicle was compressed onto the coracoid. Bicortical fixation was achieved, with the inferior cortex being breached by 2 threads of the screw.

**Figure 5:**
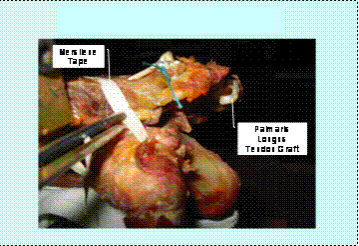
Palmaris-Longus tendon reconstruction – Mersilene tape augmentation.

• Palmaris Longus tendon – Mersilene tape augmentation (Fig 5).

Palmaris Longus tendon grafts were prepared after being harvested from the volar aspect of cadaveric forearms via two 1-cm transverse mid-axial incisions spaced about 10 cm apart. Prior to testing, a tendon graft was then passed through the 3.2 mm holes, each drilled at the distal end of the clavicle and at the acromion, 1 cm away from the acromioclavicular joint with the ends secured in a pulvertaft fashion, using No.2 Ethibond sutures, This reconstruction was reinforced with a Mersilene tape which was passed beneath the coracoid process, swung and tied on the superior aspect of the distal third of the clavicle.

Load-displacement values were analyzed for each test to determine structural properties, that is, load to failure (in newtons), stiffness (in newtons per millimeter) and displacement at failure load (in millimeters). The load to failure and displacement at failure represents the load and point at which the native ligaments fail completely. These results were recorded directly from the computer. The linear stiffness was calculated by determining the slope of the line fit to the linear portion of the load-elongation curve.

Load-displacement values were plotted simultaneously. These results for the clavicle hook plate more accurately reflect the load at which the distal clavicle end fractures or acromion fractures or when the hook dislodges from the inferior surface of the acromion.

### Statistical Analysis

A one-way analysis of variance was used for multiple comparisons amongst the 5 groups, with respect to load to failure, displacement at failure and tensile stiffness. (S-PLUS statistical software 2005). The Student's paired t-test was used only for comparison between sequential testing of native ligaments and WD reconstruction in the same specimen. Unpaired specimens were analyzed using Student's unpaired t-test. A p-value of 0.05 was used to denote the level of significance.

## Results

The loads at failure, stiffness, displacement and modes of failure for the intact ligaments and various reconstructive methods, in the superior and posterior-anterior loadings, are summarized in Table 1. The results are expressed in (Mean +/- S.E.) and (Lower and upper confidence limits – LCL, UCL) [See Additional File 2 for Boxplots 1 to 6].

**Table 1:** Comparison of the biomechanical characteristics of the intact ligaments, various reconstruction and augmentation methods

**Characteristic**	**Native**	**WD**	**WD.ACJ**	**WD.BS**	**WD.CP**	**WD.PLmt**
**Failure Loads (Superior) (kN)**	.801+/-.076	.118+/-.023	.161+/-.019	.573+/-.088	.397+/-.046	.276+/-.046
**(LCL, UCL)**	(.648, .954)	(.071, .166)	(.119, .204)	(.385, .760)	(.304, .490)	(.168, .384)
**Failure Loads (P-A) (kN)**	.746+/-.089	.103+/-.015	.278+/-.074			.188+/-.017
**(LCL, UCL)**	(.529, .963)	(.067, .139)	(.097, .459)			(.148, .229)
**Stiffness (Superior) (kN/mm)**	.079+/-.009	.006+/-.000	.015+/-.001)	.121+/-.016	.025+/-.003	.016+/-.002
**(LCL, UCL)**	(.059, .100)	(.005, .008)	(.012, .018)	(.084,.157)	(.017, .032)	(.010, .021)
**Stiffness (P-A) (kN/mm)**	.022+/-.004	.004+/-.000	.042+/-.016			.012+/-.000
**(LCL, UCL)**	(.012, .031)	(.002, .005)	(.003,.080)			(.010, .013)
**Displacement (Superior) (mm)**	25.25+/-1.77	28.70+/-1.93	29.43+/-2.63	21.92+/-4.11	26.61+/-1.26	31.16+/-3.45
**(LCL, UCL)**	(21.70, 28.81)	(24.73, 32.67)	(23.67, 35.16)	(13.06, 13.80)	(24.05,29.18)	(23.02, 39.31)
**Displacement (P-A) (mm)**	56.36+/-9.80	43.05+/-5.46	38.65+/-7.48			41.46+/-4.63
**(LCL, UCL)**	(32.38, 80.33)	(29.69, 56.40)	(20.343, 56.95)			(30.14, 52.77)
**Failure Modes**	Midsubstance	Suture failure	Suture pullout	Screw pullout	clavicle	suture
	tear 90%	at knot-clavicle	90%	100%	fracture 70%	breakage 10%
	Ligament insertion site	interface	breakage		acromion	knot
	failure 10%	100%	10%		fracture 30%	breakage 90%
(LCL, UCL)-lower and upper confidence limit						

### Load at Failure

In superior loading (Boxplot 1), the tensile strength was greatest for the native ligaments when compared to other reconstruction/augmentation (p < 0.01), but it was not significantly different from WD+BS (p = 0.10). There was, however, no significant difference in tensile strength between WD and WD.ACJ reconstruction (p = 0.26). WD-PLmt was found not to be significantly different from WD.CP (p = 0.23). WD.CP was also not significantly different from WD.BS in tensile strength (p = 0.06) but significantly stronger than WD.ACJ (p < 0.01).

In posterior-anterior loading (Boxplot 2), the native ligaments were the strongest (p < 0.01) while the WD was the weakest amongst the comparison groups (p < 0.05). Contrary to superior loading, WD.ACJ in posterior-anterior loading was significantly stronger than WD (p = 0.04). There was no significant difference in tensile strength between WD.ACJ and WD.PLmt (p = 0.26).

### Stiffness at Failure

In superior loading (Boxplot 3), the native ligaments were significantly less stiff than WD.BS (p = 0.03) but significantly stiffer than other reconstructions (p < 0.001). The WD is the least stiff (p < 0.01). No significant difference in stiffness was observed between WD.CP and WD.PLmt (p = 0.07). WD.ACJ was also not significantly stiffer than WD.PLmt (p = 0.75).

In posterior-anterior loading (Boxplot 4), the native ligaments were significantly stiffer than WD and WD.PLmt (p < 0.05). However, there is no significant difference between the native ligaments and WD.ACJ (p = 0.25). WD.ACJ and WD.PLmt were both significantly stiffer than WD alone (p < 0.05). However there was no statistical difference in stiffness between WD.ACJ and WD.PLmt (p = 0.08).

### Displacement at Failure

In both superior (Boxplot 5) and posterior-anterior (Boxplot 6) loading, there was no significant difference amongst all the comparison groups. (p > 0.05)

### Modes of Failure

The native ligaments failed at midsubstance (90%) and at the ligament insertion site (10%). In coracoacromial ligament transfer, all sutures failed at knot-clavicle interface. Suture pull-out (90%) and breakage (10%) were observed for WD.ACJ reconstruction. All coracoclavicular screws failed by screw pull-out. WD.PLmt reconstruction failed by suture (10%) or knot breakage (90%).

Most of clavicle hook plate failures occurred at 3 sites: 1) acromion fractures which occurs within 20 mm of the acromion tip, 2) distal clavicle fractures which occured at the site of the anterolateral screw holes of the clavicle hook plate and 3) the gradual deformation of the acromion in the superior direction allowed the hook of the plate to bend and slip superiorly, especially when the lateral ends of the plate have not been pre-contoured. There were no coracoid fractures as reported by Costic et al. [5].

## Discussion

This is the first study looking at the reconstruction of the acromioclavicular capsuloligamentous complex using the palmaris longus tendon graft. Biomechanical testing showed that in superior loading, it is as strong in tensile strength and as stiff as the clavicle hook plate in providing superior stability. In posterior-anterior loading, it is as strong and stiff as the ACJ capsuloligamentous repair.

Our study looks at the combined effect of native acromioclavicular and coracoclavicular ligaments, in contrast to other studies [9,17,24,26], which more closely resemble clinical situations where impact forces do not selectively damage either of these ligaments. A combined injury of both these ligaments is required to give a Rockwood type III or more severe ACJ dislocation. Double-bundle reconstitution of the conoid and trapezoid ligaments in Mazzocca's study is innovative[21], however, AC capsuloligametous repair was not mentioned and testing in the posterior-anterior direction was not performed. In this study, we have shown the pivotal role of the AC capsuloligamentous complex in providing posterior-anterior stability; however, superior stability is provided by either plate or screw augmentation or tendon graft reconstruction. Debski et al also showed that the ACJ capsule confers posterior-anterior stability and the intact coracoclavicular ligament cannot compensate for loss of capsular function during posterior-anterior loading. Failure to augment a coracoclavicular reconstruction will subject the latter to higher risk of failure. Any residual posterior-anterior instability can cause postoperative pain [6].

Weaver-Dunn reconstruction alone with coracoacromial ligament is insufficient. Incomplete reduction or recurrence of dislocation was reported to be as high as 24% [27]. We found its strength to be one-eighth that of the native combined AC+CC ligaments (801 +/- 75) N. Harris et al reported its strength to be one-quarter that of CC ligaments (500+/-134) N alone. Various augmentation methods have been described. [14] Although none of the augmentative methods tested restored acromioclavicular stability to normal, all proved superior to the Weaver-Dunn reconstruction alone [7]. In addition, Deshmukh et al showed that, the contribution of Weaver-Dunn transfer to the stability, when combined with augmentative fixation, is negligible at time zero. This further justifies for the need for augmentation.

We found that both BS and clavicle hook plate devices provide adequate augmentation. BS provided 70% and 170% of the tensile strength and stiffness of the native ligaments, respectively. On the other hand, clavicle hook plate provided 50% and 30% of the tensile strength and stiffness of the native ligaments, respectively. The results of BS augmentation are consistent with that reported by Motamedi et al. [22]. Urist found that failure strength, however, was reduced by half if only unicortical purchase was obtained, indicating the importance of accurate screw placement [27]. Disadvantages of this screw fixation technique include complications during screw insertion, screw irritation, infection, pullout and breakage [14]. Early deformity recurrence may occur with early implant removal.

An ideal augmentation device should, biomechanically, have a similar compliance as that of native ligaments. Too stiff a device can predispose to bone breakage and cause joint stiffness ex vivo, whilst too compliant a device can cause premature failure of the Weaver-Dunn construct during rehabilitation. Distal clavicle resection as part of Weaver-Dunn reconstruction, described by Mumford, was thought to prevent postoperative pain and osteolysis [23]. This was shown not to be the case by Browne JE [3]. We found that distal clavisectomy precludes ACJ repair and prevents proper seating of the clavicle hook plate.

Several considerations need to be made when using the clavicle hook plate. Further prebending of the plate may be required to allow optimal sitting on the clavicle. The narrow rectangular-shaped clavicle hook under the acromion surface causes tremendous contact stress and predisposes to acromial fracture during loading. A more rounded disk-shaped anchorage will be ideal. Extreme care must be exercised during the drilling of the anterolateral distal holes since stress fractures have occurred at these sites. Insertion of medial screws may be sufficient. Distal resection of the distal clavicle or the use of autogenous grafts such as semitendinosus or palmaris longus graft for the reconstruction of the acromioclavicular capsuloligamentous complex will preclude the use of clavicle hook plates because of inadequate sitting of the implant on the clavicle. A further consideration ex vivo is that of subacromial impingement which will need to be explored in post-operative patients. The need for implant removal following graft incorporation, as with the coracoclavicular screw fixation, is a disadvantage compared to autologous grafts or biodegradable substances such as Mersilene tapes. The current clavicle hook plate does not address posterior-anterior instability and translation of the acromoclavicular joint. A routine repair or reconstruction of the acromioclavicular joint capsuloligamentous complex can address this problem.

Coracoclavicular ligament reconstruction using tendon grafts have been widely described. There are the advantages of biological integration, no fracture or loosening, no need for implant removal and low morbidity with graft harvesting.

In this study, we used the palmaris longus tendon graft, in addition to the Weaver-Dunn procedure, to reconstruct the acromioclavicular capsuloligamentous complex and augmented it with a 5 mm Mersilene tape which looped around the coracoid process and clavicle. The tendon graft may benefit from augmentation with the tape to protect the repair, limit the amount of possible stretching and counteract the weakening effects of revascularization. This reconstruction had tensile strength not significantly different from the clavicle hook plate with superior loading and similar to ACJ capsuloligamentous repair with posterior-anterior loading. We also noticed that its flat cross-sectional area, superior-inferiorly, also helped in its sitting across the acromioclavicular joint. It therefore served a dual function of stabilizing the acromioclavicular joint in both the posterior-anterior and superior directions while protecting the concurrent WD reconstruction. The palmaris longus tendon grafts used here for ACJ ligament reconstruction were about 10 cm long, as opposed to the 16 cm palmaris longus graft used by Lee et al for coracoclavicular ligament reconstruction [19].

Grutter and Petersen showed that, when tested in coronal plane only, the Weaver-Dunn reconstruction, palmaris-longus tendon graft and flexor carpi radialis graft achieve tensile strength 59%, 40% and 95% that of the native ACJ capsule [12]. These were in contrast to our findings, with the WD and palmaris longus reconstruction achieving, in the coronal plane, 14.7% and 34% that of the combined native ligaments and in the sagittal plane, 8% and 19.6% that of the combined native ligaments. The discrepancy in results arose because Grutter et al compared the tensile strength of the reconstruction with that of the native ACJ capsule only (simulating grade II and below injury) whereas we compared the tensile strength of our reconstruction with that of the combined native AC + CC ligaments (simulating grade III and above injury).

Suture failures were noted in WD reconstruction, ACJ repair and PL-mt reconstruction. This was not surprising given the fact that the sutures were weaker in tensile strength compared to that of the CAL, ACL or palmaris longus ligament, as shown by Harris et al. [17] and Lee et al. [21]. Native ligaments failed at mid-substance at the low strain rate used in our study. However, most specimens will have bony avulsion if high strain rates are used. Therefore, the crosshead speed or strain rate must be specified to suit the purpose of one's study. For clavicle hook plates testing, the probability of a clavicle fracture is dependent on the bone size and amount of bone bridge in between drill holes. On the other hand, a strong fixation on the clavicle will result in plate failure by acromial deformation or fracture.

The strengths of the current study were observed. Firstly, baseline tensile strength results of the native ligaments were made available for comparison with other reconstructive and augmentative groups, in both coronal and sagittal planes. Secondly, the testing setup has 3 degrees of freedom and allows firm hold on the scapular blade and proximal clavicle. It allows plastic bending of the acromion, coracoid process and distal clavicle, which further simulates a real-event injury. In-situ precise testing of native ligaments and sequential repair or augmentation were performed without removing the specimens from the testing apparatus [18,19]. Thirdly, the methods used to measure failure load and failure displacement for each group were precise, objective and reproducible. Loads to failure were consistently applied in both coronal and sagittal directions for various conditions tested in each specimen because the specimens were not removed from the test rig when the reconstruction or augmentation procedures were performed. Fourthly, reconstructive and augmentative procedures were performed in conjunction with the modified Weaver-Dunn procedure and finally, the failure loads of the native ligaments were measured in comparison with the capsuloligamentous reconstruction and the various augmentative repairs. The palmaris-longus tendon graft-mersilene tape graft was tested uniquely in reconstructing the ACJ, in contrast to other studies where it was used to reconstruct the CC ligament.

Concurrently, a few limitations of this study were also seen. Firstly, tensile loading was performed only in the superior axis at a much lower strain rate than that which would have occurred during injury. Secondly, repetitive testing on a bony specimen may cause plastic deformation of the clavicle and acromion and predispose to bony failure in some specimens; conversely, the ligament repair and reconstruction were performed on uninjured joints and did not account for any damage to the coracoid or clavicle which may accompany the injury. Thirdly, the cyclic and static viscoelastic properties of the native ligaments and fatigue properties of the clavicle hook plate and coracoclavicular screw, have not been determined. Finally, it has been shown that all of the soft tissues at the acromioclavicular joint function synergistically, in a complex manner, to provide joint stability. Thus, traumatic disruption of the acromioclavicular joint capsule is thought to result in abnormal joint kinematics and load transmission, factors that increase the possibility of postinjury pain, instability, and degenerative joint disease. These are factors which could not be tested in this study [8,27].

## Conclusion

Modified Weaver-Dunn procedure must always be supplemented with acromioclavicular capsuloligamentous repair to increase posterior-anterior stability. Palmaris-Longus tendon graft provides both additional superior and posterior-anterior stability when used for acromioclavicular capsuloligamentous reconstruction. It is therefore a good alternative to clavicle hook plate in acromioclavicular injuries.

## Authors' contributions

GEL dissected the specimens, designed the methodology, conducted the experiments, performed the statistical analysis and drafted the manuscript.

CKY was involved in the conception, participated in the coordination of the study and data analysis.

DAS and SS were involved in the critical revision of the manuscript.

DSK was involved in conceptual input to the study.

All authors read and approved the final manuscript.

## Supplementary Material

Additional file 1 Graph showing the centroid of the acromion. Click here for file

Additional file 2 Boxplots showing results of loads, stiffnesses and displacements at failure, in the superior and posterior-anterior directions of the various augmentative and reconstructive methods. Click here for file

## References

[B1] Bargren JH, Erlanger S, Dick HM (1978). Biomechanics and comparison of two operative methods of treatment of complete acromioclavicular separation. Clin Orthop.

[B2] Bosworth BM (1941). Acromioclavicular separation. A new method of repair. Surg Gynecol Obstet.

[B3] Browne JE (1977). Acromioclavicular joint dislocations. Comparative results following operative treatment with and without clavisectomy. Am J Sports Med.

[B4] Cadenat FM (1917). The treatment of dislocations and fractures of the outer end of the clavicle. Int Clin.

[B5] Costic R, Labriola J, Rodosky M (2004). Biomechanical rationale for development of anatomical reconstructions of coracoclavicular ligaments after complete acromioclavicular joint dislocations. Am J Sports Med.

[B6] Crowinshield RD, Pope MH (1976). The strength and failure rat medial collateral ligament. Journal of Trauma.

[B7] Debski RE, Parsons IM, Woo Savio L-Y, Fu Freddie H (2001). Effect of capsular injury on acromioclavicular joint mechanics. JBJS(American Volume).

[B8] Deshmukh AV, Wilson D, Zilberfarb J (2004). Stability of acromioclavicular joint reconstruction-Biomechanical testing of various surgical techniques in a cadaveric Model. Am J Sports Med.

[B9] Dorlot (1980). Load elongation behavior of canine anterior cruciate ligament. J Biomech Eng.

[B10] Dumontier C, Sautet A, Man M (1995). Acromioclavicular dislocations: treatment by coracoacromial ligamentoplasty. J Shoulder Elbow Surg.

[B11] Flatow EL (1993). The biomechanics of the acromioclavicular, sternoclavicular, and scapulothoracic joints. Instr Course Lect.

[B12] Fukuda K, Craig E, An KN, Cofield RH, Chao EY (1986). Biomechanical study of the ligamentous system of the acromioclavicular joint. J Bone Joint Surg Am.

[B13] Galpin RD, Hawkins RJ, Grainger RW (1985). A comparative analysis of operative versus nonoperative treatment of grade III acromioclavicular separations. Clin Orthop.

[B14] Grana WA, Egle DM, Mahnkan R (1994). An analysis of autograft fixation after anterior cruciate ligament reconstruction in a rabbit model. Am J Sports Med.

[B15] Grutter PW, Petersen SA (2005). Anatomical ACJ reconstruction: A biomechanical comparison of reconstructive techniques of acromioclavicular joint reconstruction. Am J Sports Med.

[B16] Guy DK, Wirth MA, Griffin JL (1998). Reconstruction of chronic and complete dislocations of the acromioclavicular joint. Clin Orthop.

[B17] Harris R, Wallace A, Harper G (2000). Structural properties of the intact and the reconstructed coracoclavicular complex. Am J Sports Med.

[B18] Hessmann M, Gotzen L, Gehling H (1995). Acromioclavicular reconstruction augmented with polydioxanonsulphate bands: surgical technique and results. Am J Sports Med.

[B19] Klimkiewicz JJ, Williams GR, Sher JS (1999). The acromioclavicular capsule as a restraint to posterior translation of the clavicle: a biomechanical analysis. J Shoulder Elbow Surg.

[B20] Lancaster S, Horowitz M, Alonso J (1987). Complete acromioclavicular separations: a comparison of operative methods. Clin Orthop.

[B21] Lee KW, Debski RE, Chen CH (1997). Functional evaluation of the ligaments at the acroimioclavicular joint during anteroposterior and superoinferior translation. Am J Sports Med.

[B22] Lee SJ, Nicholas SJ, Akizuki KH, McHugh MP, Kremenic IJ, Ben-Avi S (2003). Reconstruction of the coracoclavicular ligaments with tendon grafts: a comparative biomechanical study. Am J Sports Medicine.

[B23] Lemos M (1998). The evaluation and treatment of the injured acromioclavicular joint in athletes. Am J Sports Med.

[B24] Magen HE (1999). Structural properties of 6 tibial fixation methods for ACL soft tissue grafts. Am J Sports Med.

[B25] Mazzocca AD, Santangelo SA, Johnson ST, Rios CG, Dumonski ML, Arciero RA (2006). A biomechanical evaluation of an anatomical coracoclavicular ligament reconstruction. Am J Sports Med.

[B26] Motamedi A, Blevins F, Willis M (2000). Biomechanics in the coracoclavicular ligament complex and augmentations used in its repair and reconstruction. Am J Sports Med.

[B27] Mumford EB (1941). Acromioclavicular dislocation. J Bone Joint Surg Am.

[B28] Neviaser JS (1968). Acromioclavicular dislocation treated by transference of the coraco-acromial ligament. a long-term follow-up in a series of 112 cases. Clin Orthop.

[B29] Nordin M, Frankel VH (1980). Basic Biomechanics of the Musculoskeletal System.

[B30] Noyes FR (1974). Biomechanics of ACL failure: an analysis of strain-rate sensitivity and mechanism of failure in primates. J Bone Joint Surg.

[B31] Noyes FR (1976). The strength of the anterior cruciate ligament in humans and rhesus monkeys. J Bone Joint Surg.

[B32] Rockwood CA, Williams GR, Young DC, Rockwood CA Jr, Green DP Bucholz RW, Heckman JD (1996). Injuries to the acromioclavicular joint. Rockwood and Green's fractures in adults.

[B33] Rodeo SA, Arnoczky SP, Torzilli PA, Hidaka C, Warren RF (1993). Tendon-healing in a bone tunnel. A biomechanical and histological study in the dog. J Bone Joint Surg Am.

[B34] Salter EG, Nasca RC, Shelly BS (1987). Anatomical observations on the acromioclavicular joint and supporting ligaments. Am J Sports Medicine.

[B35] Ticker JB (1996). Inferior glenohumeral ligament geometric and strain-rate dependent properties. J Shoulder and Elbow Surg.

[B36] Urist MR (1946). Complete dislocations of the acromioclavicular joint. The nature of the traumatic lesion and effective methods of treatment with an analysis of forty-one cases. J Bone Joint Surg.

[B37] Wamis Singhatat (2002). How four weeks of implantation affect the strength and stiffness of a tendon graft in bone tunnel: a study of 2 fixation devices in an extraarticular model in ovine. Am J Sports Med.

[B38] Weaver J, Dunn H (1972). Treatment of acromioclavicular injuries, especially complete acromioclavicular separation. J Bone Joint Surg Am.

[B39] Weiler A (1998). Hamstring tendon fixation using interference screws: A biomechanical study in calf tibial bone. Arthroscopy.

[B40] Weinstein D, McCann P, McIllveen S (1995). Surgical treatment of complete acromioclavicular dislocations. Am J Sports Med.

[B41] Woo SL (1987). Effect of knee flexion on structural properties of rabbit femur-ACL-tibia complex. Journal of Biomechanics.

[B42] Woo SL (1990). The effect of strain rate on properties of the medial collateral ligament in skeletal immature and mature rabbits: a biomechanical and histological study. J of Orthop Research.

[B43] Woo SL, Debski RE, Withow JB (1999). Biomechanics of Knee Ligaments. Am J Sports Med.

