# Supplementary figures and images for: Acromioclavicular joint dislocation: a comparative biomechanical study of the palmaris-longus tendon graft reconstruction with other augmentative methods in cadaveric models
Source: J Orthop Surg Res. 2007 Nov 27;2:22. doi: 10.1186/1749-799X-2-22 (PMC2235831; doi:10.1186/1749-799X-2-22)

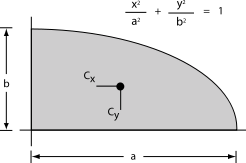

Supplement: Additional file 1 — Graph showing the centroid of the acromion. [file 1749-799X-2-22-S1.doc]
