# Supplementary material for: Acromioclavicular joint dislocation: a comparative biomechanical study of the palmaris-longus tendon graft reconstruction with other augmentative methods in cadaveric models
Source: J Orthop Surg Res. 2007 Nov 27;2:22. doi: 10.1186/1749-799X-2-22 (PMC2235831; doi:10.1186/1749-799X-2-22)
Supplement: Additional file 2 — Boxplots showing results of loads, stiffnesses and displacements at failure, in the superior and posterior-anterior directions of the various augmentative and reconstructive methods. [file 1749-799X-2-22-S2.doc]

Loads at Failure


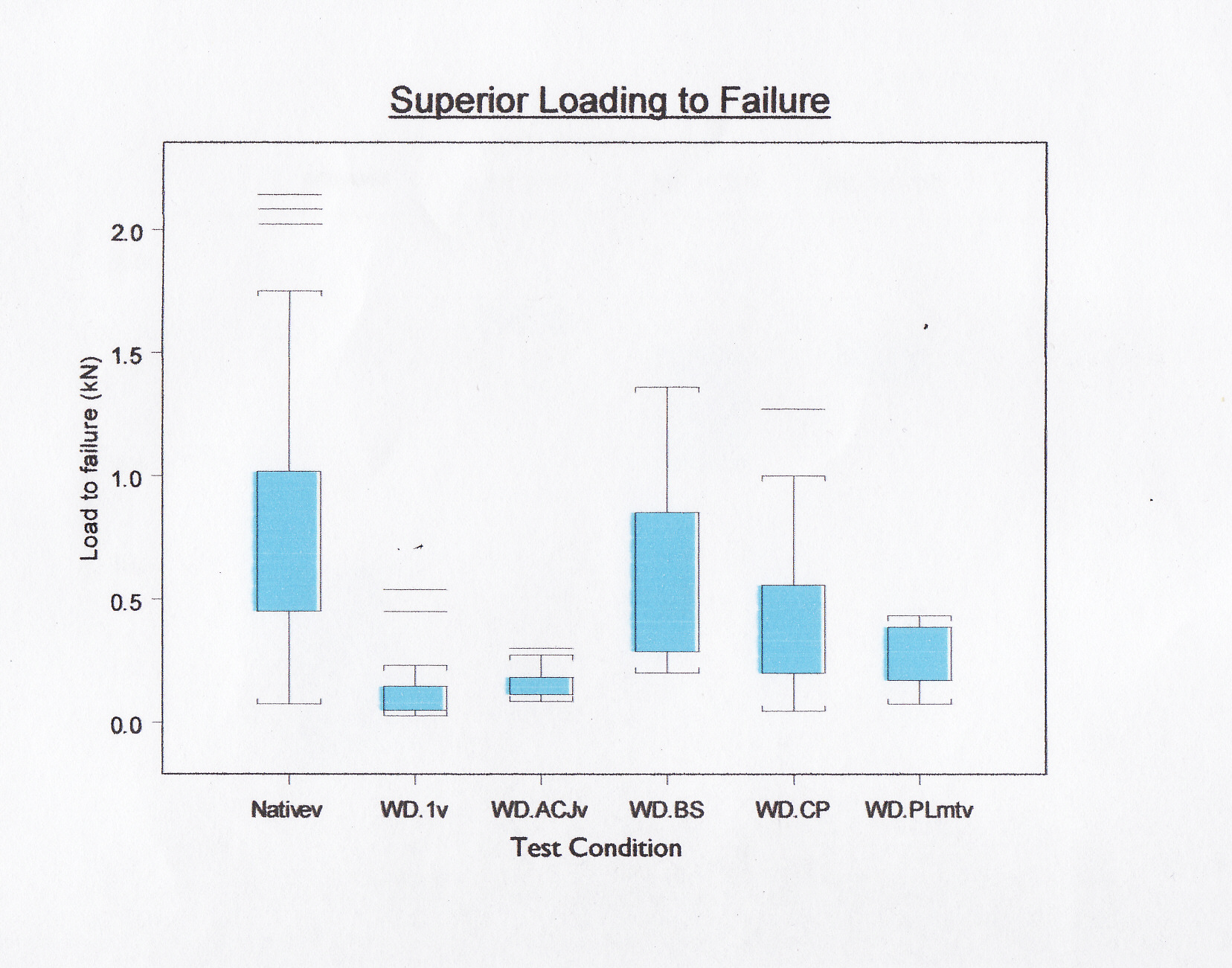


Boxplot 1. Result showing loads to failure in the superior direction for the native

ligaments and various reconstruction methods


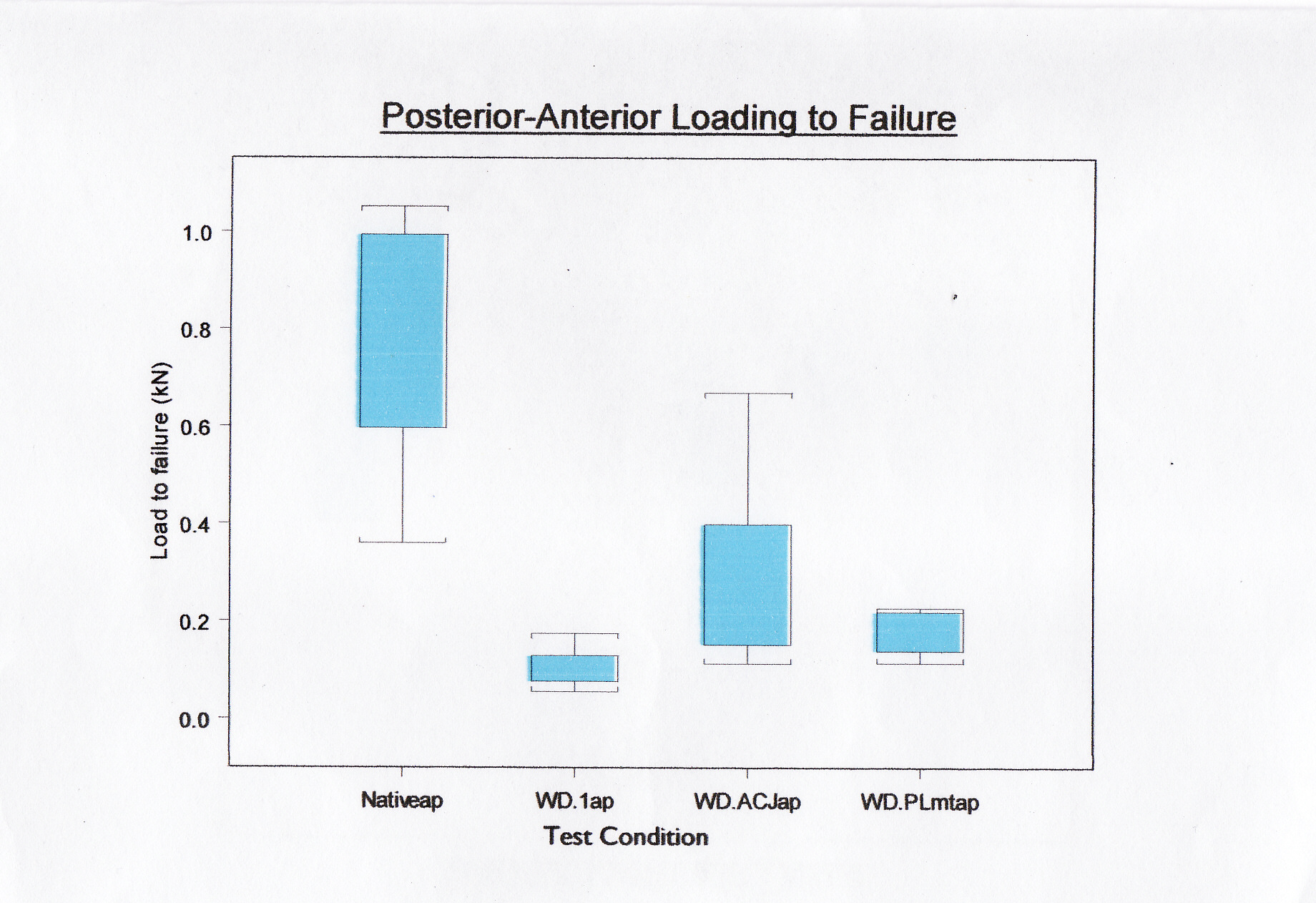


Boxplot 2. Result showing loads to failure in the posterior-anterior direction for the native ligaments and various reconstruction methods

Stiffnesses at Failure


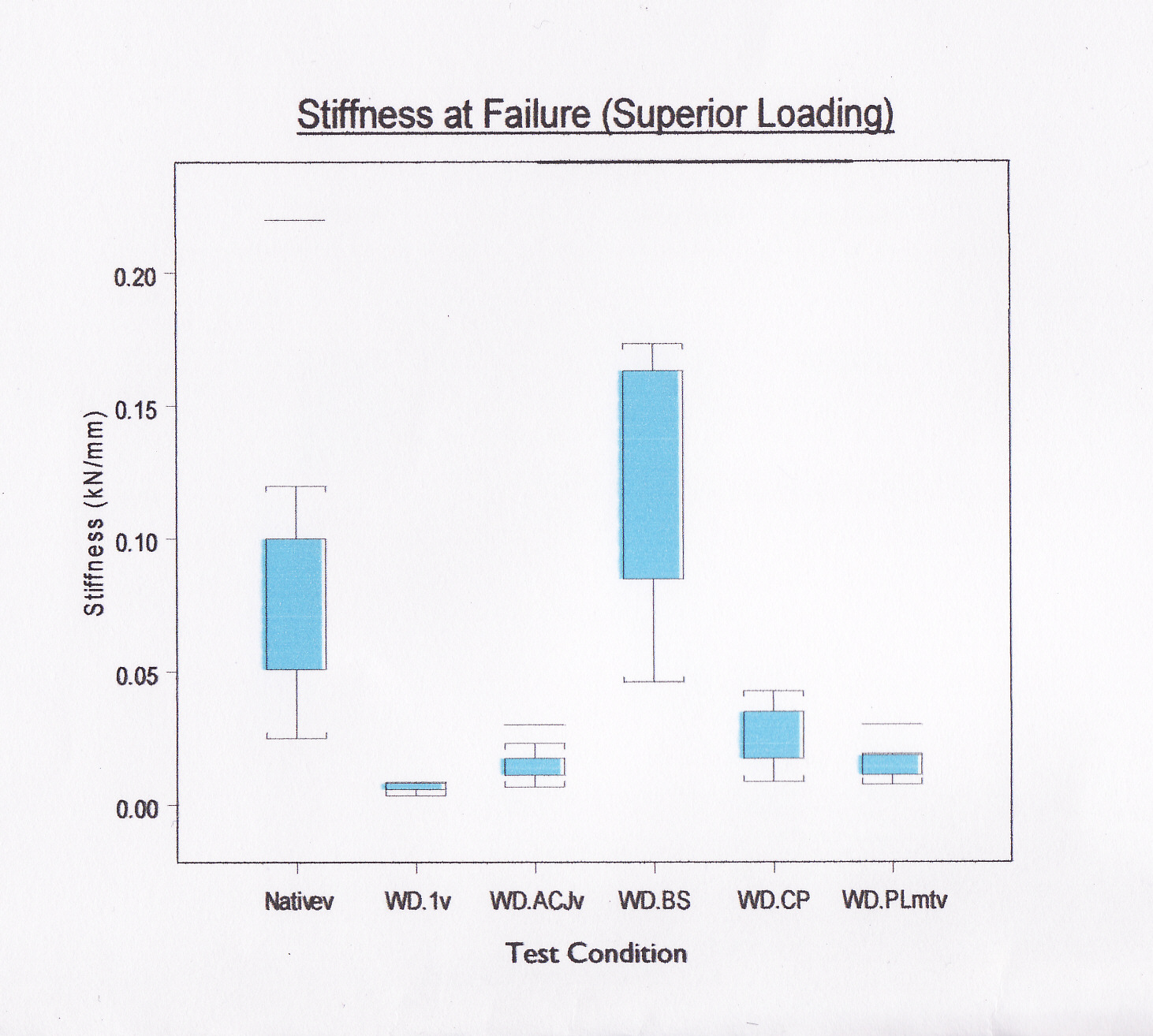


Boxplot 3. Result showing stiffness at failure in the superior direction for the native ligaments and various reconstruction methods


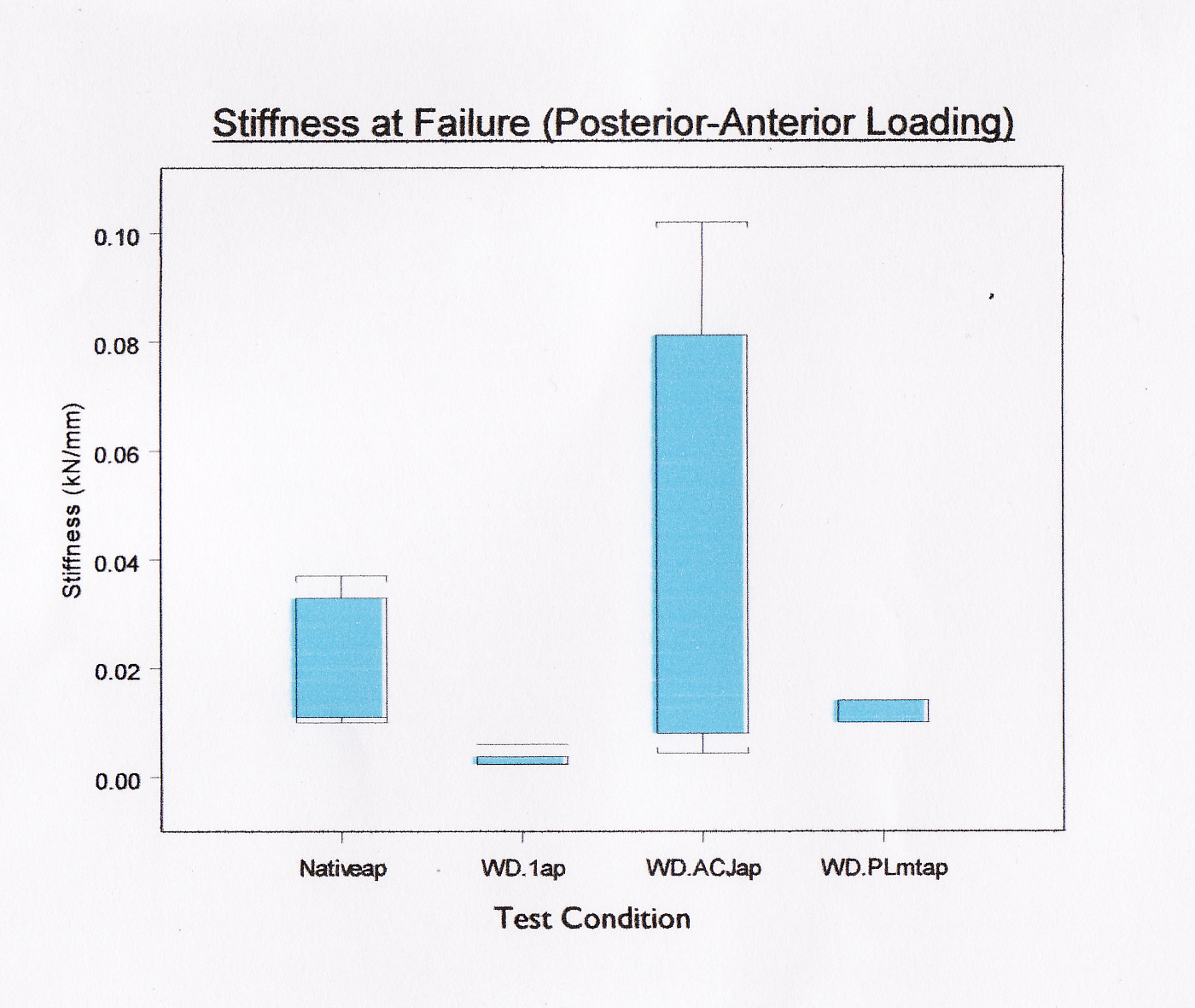


Boxplot 4. Result showing stiffness at failure in the posterior-anterior direction for the native ligaments and various reconstruction methods

Displacement at Failure


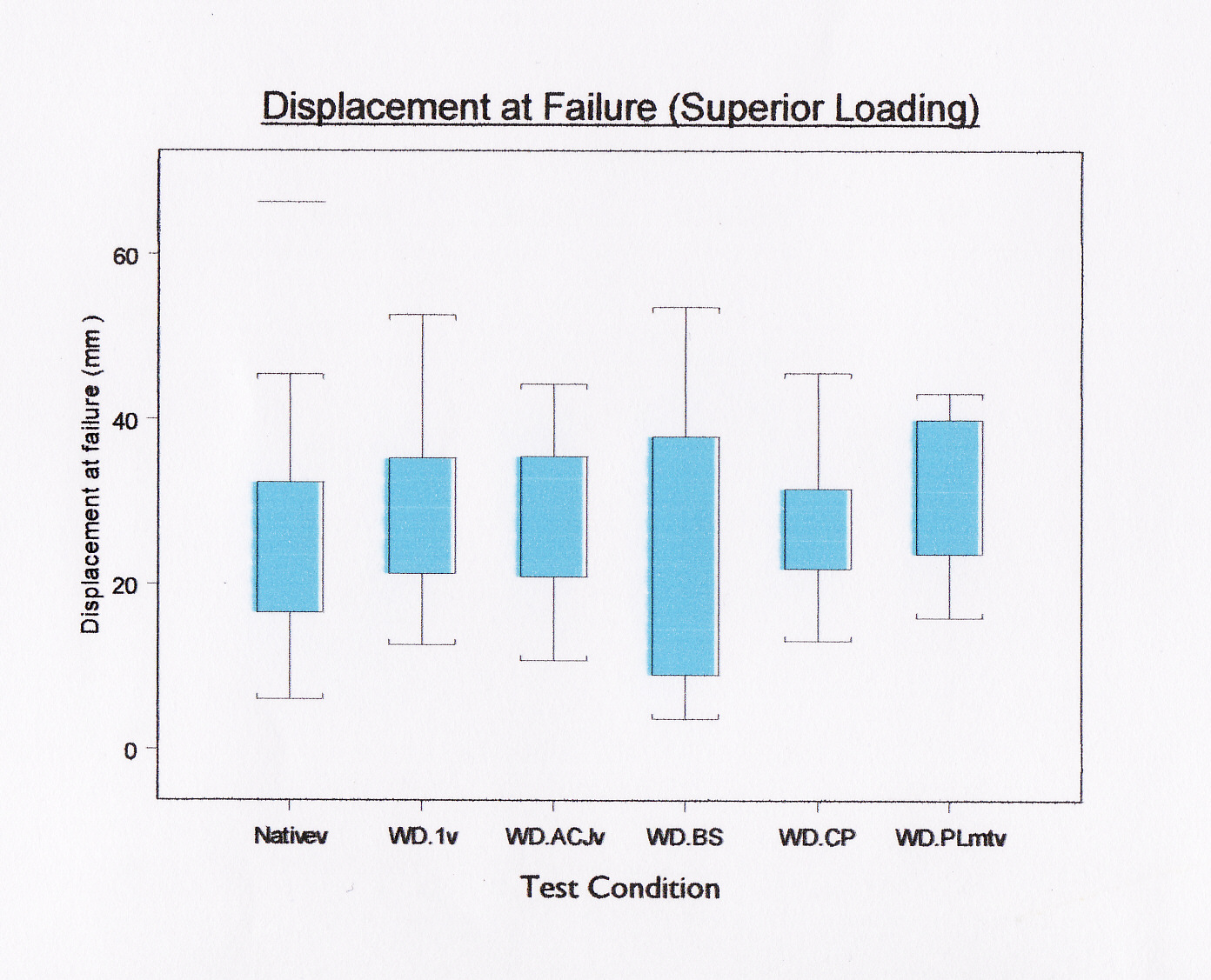


Boxplot 5. Result showing displacement at failure in the superior direction for the native ligaments and various reconstruction methods


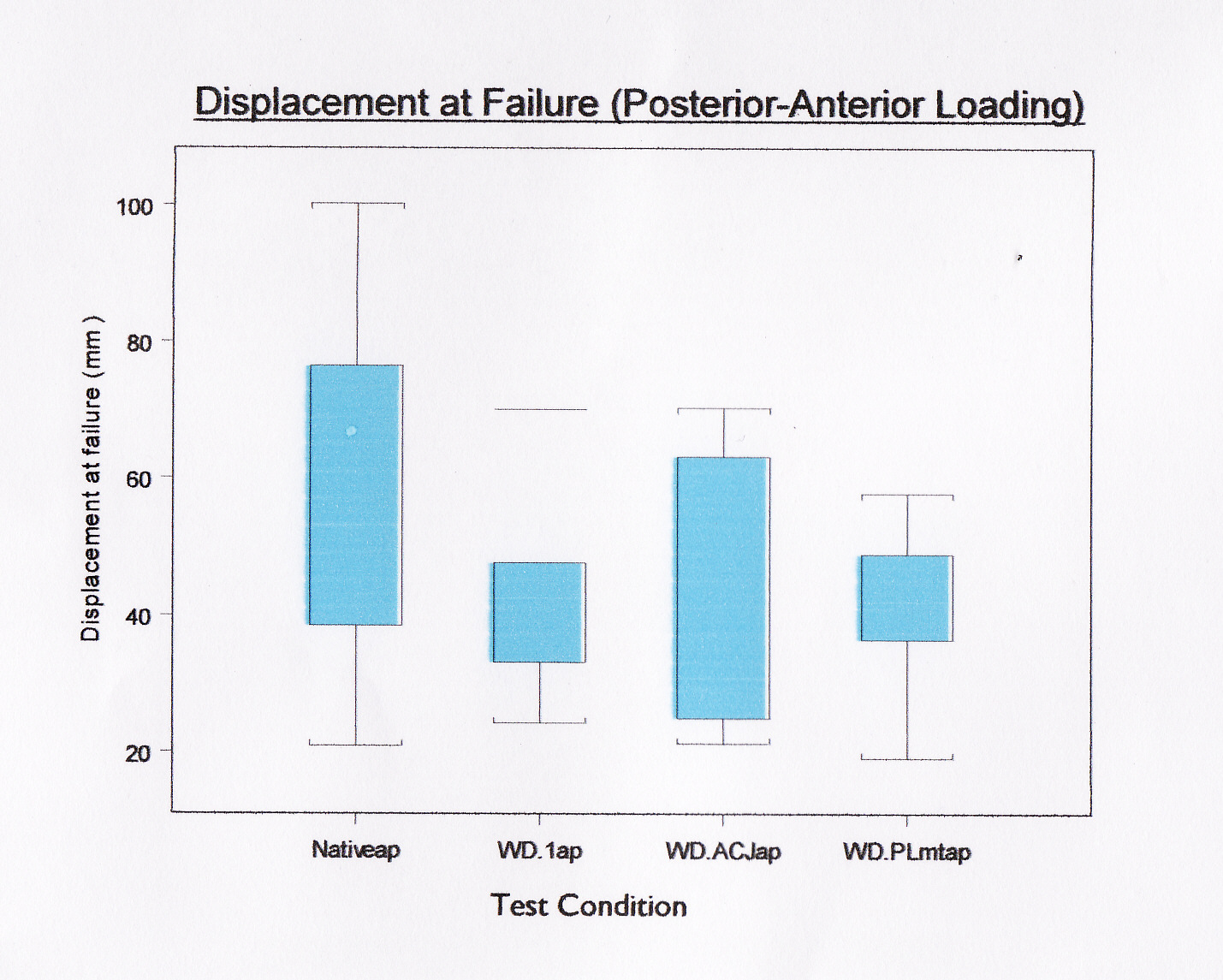


Boxplot 6. Result showing displacement at failure in the posterior-anterior direction for the native ligaments and various reconstruction methods
